# Practical Steps to Advance Health Equity in Research from the UCLA COVID-19 Health Equity Research and Advisory Committee

**DOI:** 10.1089/heq.2024.0088

**Published:** 2024-09-17

**Authors:** Enrico G. Castillo, Alma D. Guerrero, Eraka P. J. Bath, Rochelle A. Dicker, Eric Esrailian, Helena Hansen, Nina T. Harawa, Breena R. Taira, Christina Harris

**Affiliations:** ^1^ Department of Psychiatry and Biobehavioral Sciences, David Geffen School of Medicine, Jane and Terry Semel Institute for Neuroscience and Human Behavior at UCLA, UCLA, Los Angeles, California, USA.; ^2^ David Geffen School of Medicine, UCLA Mattel Children’s Hospital, UCLA, Los Angeles, California, USA.; ^3^ Department of Surgery, University of California San Francisco, San Francisco, California, USA.; ^4^ Vatche and Tamar Manoukian Division of Digestive Diseases, David Geffen School of Medicine, UCLA, Los Angeles, California, USA.; ^5^ Division of General Internal Medicine & Health Services Research, David Geffen School of Medicine at University of California Los Angeles, Los Angeles, California, USA.; ^6^ Department of Psychiatry, Charles R. Drew University of Medicine and Science, Los Angeles, California, USA.; ^7^ Department of Emergency Medicine, Olive View-UCLA Medical Center, Sylmar, California, USA.; ^8^ David Geffen School of Medicine, UCLA, Los Angeles, California, USA.; ^9^ Department of Medicine, Cedars-Sinai Medical Center, Los Angeles, California, USA.

**Keywords:** research, health equity, medical research, community, diversity and inclusion

## Abstract

**Introduction:**

The University of California, Los Angeles COVID-19 Health Equity Research and Advisory Committee was created to identify and fund research to address COVID-19 inequities.

**Methods:**

The committee addressed barriers to health equity research and funded $1.5 million of research.

**Results:**

These actions facilitated dialogue, shifted research infrastructure, and piloted strategies to enhance health equity impacts through consultation and feedback. We provide an overview of projects funded and highlight one to demonstrate impact.

**Conclusion:**

We provide a framework to help institutions implement similar approaches to centering health equity in research.

## Introduction

Minoritized and under-resourced communities are too often excluded from the latest innovations and benefits of biomedical research.^1–4^ Early in the COVID-19 pandemic, Los Angeles saw twice the rate of death among Black and Latiné individuals compared with White individuals, and four times the rate of death in high poverty communities.^5^ The director of the Los Angeles County Department of Public Health said, “These results are absolutely devastating and represent real people whose lives have been lost. They also starkly show how inequities have a life and death consequence.”^5^

The University of California, Los Angeles (UCLA) sought to address local inequities through its emerging portfolio of COVID-19 research. In May 2020, UCLA’s School of Medicine convened the COVID-19 Health Equity Research and Advisory Committee to identify and fund research that would directly address local pandemic-related inequities. Between May 2020 and April 2021, our committee fundraised and administered $1.5 million in research funding to 16 teams. Our committee also created and published the Health Equity Research Impact Assessment (HERIA), a tool to help achieve a more equitable research ecosystem by encouraging critical reflexivity about health equity.^1^ HERIA’s development and domains are described in detail in a prior publication.^1^

This Innovation Report will focus on generalizable lessons drawn from the experience of this committee to help advance health equity within biomedical research. Here, we describe our successful engagement with researchers and university leadership to facilitate health equity research partnerships and generate funding through targeted fundraising, examples of local impacts, and the reach of this work domestically and internationally.

## Seven Structural Actions to Promote Health Equity Research

The COVID-19 Health Equity Research and Advisory Committee was created to identify and fund biomedical research studies on COVID-19-related health and social inequities. The committee was comprised of nine researchers in emergency medicine, epidemiology, gastroenterology, internal medicine, pediatrics, psychiatry, and surgery. There was an intentional effort by the co-chairs for the membership of the committee to reflect Los Angeles’ rich racial and ethnic diversity. Members brought expertise in public-academic-community partnered research and a deep understanding of health disparities research frameworks and strategies. Committee members understood that actions at multiple levels throughout the university research infrastructure and local community were needed to advance health equity-focused COVID-19 research, the committee conducted seminars with basic science, translational, and clinical research committees; university development officers; and university research leadership, taking care to spotlight local pandemic-related inequities and obstacles that health equity researchers faced at the university. This outreach led to philanthropic support for COVID-19 health equity research and to reforms in university research policies and practices.

The committee worked closely with UCLA research leadership on seven actions to address challenges faced by health equity researchers and to improve the impact of the funded research. First, funding was prioritized for projects that focused on populations disproportionately impacted by the pandemic, supported by $1.5 million in funding from philanthropy and UCLA research leadership. Second, the eligibility for this funding opportunity was expanded to include faculty and institutions that were commonly excluded from university grants, including those at affiliated UCLA campuses and the Charles R. Drew University of Medicine and Science, a historically Black medical school in South Los Angeles. Third, the committee shared the funding announcement with local networks of health equity researchers to prioritize applicants from this pool of researchers. Fourth, investigators were required to detail their plans for addressing local inequities, choosing from among health equity research priority areas that were developed and publicized by the committee (Box 1). These priority areas were in the domains of public-academic-community partnered interventions and services, policy research, health services research, education, and advocacy. Fifth, the committee required investigators to detail their plans to disseminate research findings in local, non-academic settings (e.g., schools, community centers). Sixth, eligibility criteria and review process were made transparent by publicizing our review criteria and decision-making processes and by providing individual feedback to research teams. Lastly, funding decisions were widely publicized to celebrate the work of researchers who were funded, to foster a sense of community among health equity researchers, and to promote the university’s investment in this line of research.

Box 1. Examples of COVID-19 Health Equity Research Priority Areas **Public-Academic-Community Partnered Interventions and Services**

- Pilot interventions of new health programs and new partnerships with community-based organizations and public agencies that will address COVID-19 health, healthcare, and/or social inequities and will create sustainable infrastructures for long-term service delivery
- Multi-level interventions that promote the capacity of community-based organizations to conduct COVID-19 testing, delivery supplies (e.g., PPE), and provide other supportive services to address the health, healthcare, and social inequities in under-resourced communities
- Community-based and community-partnered interventions, particularly those that operate at the level of whole communities, which address health, healthcare, and social inequities for entire communities and populations
- Innovative interventions that address data, healthcare and social service delivery, and educational outreach needs of local public safety net agencies
- Prioritize research that minimizes the “digital divide” to increase access to COVID-19 knowledge and services, increase access to primary care physicians and care during this pandemic:
- Telemedicine
- Public service announcements
- “Ask the doctor” podcasts
- Video chats
- Intervention in an UCLA Health digital venue (e.g., UCLA Health Zone radio show and podcast)

**Policy Research**

- Studies investigating the effects of COVID-19 healthcare, public health, and public policies, including UCLA and other institutional-level clinical and research policies, on health, healthcare, and social inequities experienced by racial/ethnic minority, under-resourced, and vulnerable individuals and communities

**Health Services Research to Address Inequities**

- Quality improvement research to address known UCLA and UCLA Health clinical, research, administrative and other processes to address COVID-19-related health, healthcare, and social inequities
- Mixed-methods evaluations to identify and develop solutions to COVID-19-related health, healthcare, and social inequities within UCLA research and UCLA Health clinical policies and services
- Dissemination and implementation science on evidence-based guidelines, treatments, and services for racial/ethnic minority, under-resourced, and vulnerable communities
- Mixed methods studies to investigate individual- to structural-level barriers to COVID-19 care (e.g., testing, public health surveillance, healthcare services) experienced by racial/ethnic minority, under-resourced, and vulnerable individuals and communities

**Medical Education and Advocacy**

- Development and mixed-methods evaluations of undergraduate or graduate medical education and/or advocacy interventions that contribute to DGSOM’s mission of improving health equity and directly benefit under-resourced Los Angeles communities
- Studies that incorporate residents, fellows, medical students, psychology interns, undergraduate and graduate students, and other trainees in the delivery of interventions to address COVID-19-related health, healthcare, and social inequities

## Community Impact on Pandemic-Related Inequities

The committee’s greatest impacts were through the distribution of $1.5 million in funding to 16 UCLA research teams to address local pandemic-related inequities. Eleven principal investigators were women, seven were researchers of color, with five being Black and/or Latiné. All 16 studies addressed local inequities exacerbated by the pandemic, particularly for Black, Latiné, transgender, and gender non-conforming populations. Researchers partnered with county health and social service agencies, non-profit organizations, schools, hospital systems, people with lived experiences of homelessness, and diverse communities across Los Angeles. Funded studies addressed barriers to gender-affirming care, vaccine hesitancy among unhoused and multi-ethnic communities, Spanish web-based resources on COVID-19 safe practices and vaccinations, food insecurity in medical-legal community partnerships, and the mental health and educational needs of public-school students. The study highlighted below exemplifies how the committee used the HERIA to review and fund studies aligned with our health equity priority areas, by providing feedback to researchers that enhanced their local impacts and partnerships.

Dr. Brian Cole in the UCLA Department of Environmental Health Sciences proposed a study to conduct environmental sampling to detect airborne SARS CoV-2 viral particles in Los Angeles public housing projects. The HERIA was used to review the proposal and prompted the committee to request that he revise the study to include a formal partnership with the Housing Authority of the City of Los Angeles (HACLA), to involve public housing tenants more inclusively in the research process and to create opportunities for data-driven policy change.

Dr. Cole was highly responsive and spent several months formalizing his research partnership with HACLA. At the conclusion of his project, he shared policy briefs with HACLA on recommendations to minimize COVID exposure in public housing buildings. This generated interest among HACLA’s leadership in air pollution in and around public housing units. HACLA successfully applied for a 2-year federal environmental justice grant and collaborated with Dr. Cole’s team as the project evaluator. He also developed ongoing relationships with several public housing tenants who became interested in his research and will help to train other community members for his future projects. The engagement between the committee and Dr. Cole, facilitated by the HERIA, helped to make this project more accountable to local communities and led to further funding opportunities and growing research partnerships.

## Future Directions

The COVID-19 Health Equity Research and Advisory Committee highlighted the criticality of health equity research, leading to changes at UCLA and the broader biomedical research ecosystem. The seven structural actions taken by the committee have been adopted by the UCLA Clinical and Translational Science Institute and other departmental research funding opportunities to continue to facilitate health equity-oriented research. A systematic and structured process to review health equity research is now in place at UCLA using the HERIA tool. Furthermore, the HERIA tool has been accessed online over 6100 times, cited by 37 peer-reviewed articles, and used by research funders internationally.^1,6^ Lastly, the committee piloted strategies to support researchers to enhance the health equity impacts of their studies through consultation, feedback, and dissemination activities.

The committee, however, also encountered challenges in identifying and funding research to address COVID-19 inequities. Too often, the timelines of research funding opportunities seeking to address health inequities are incompatible with the necessary processes to co-develop that research in partnership with communities most impacted by inequities. The committee experienced this challenge, noting that many qualified researchers chose not to apply due to the short application timeline. The committee found that applications that were most successful in addressing health inequities were those that already had established community partnerships in place. Additionally, while individualized feedback to research teams enhanced projects’ health equity impact, the committee found this to be an unexpectedly time-consuming effort, which would have been helped by a larger pool of health equity research reviewers. Future efforts should consider using more flexible application and research timelines and deploy larger teams of health equity reviewers to address these challenges.

Many medical researchers seek opportunities to make an impact on equity, within their institutions and in local minoritized and under-resourced communities. Health equity does not arise from one idea or one project. The future of health equity research will involve the creation of new tools; the re-imagining of research policies, practices, and incentive structures; the destruction of barriers to translational and community partnerships; and the transformation of funding priorities—to promote health equity and justice in biomedical research and our local communities.

## Abbreviations Used

HACLA: Housing Authority of the City of Los Angeles
HERIA: Health Equity Research Impact Assessment
UCLA: University of California, Los Angeles
